# Melatonin antiproliferative effects require active mitochondrial function in embryonal carcinoma cells

**DOI:** 10.18632/oncotarget.4012

**Published:** 2015-05-20

**Authors:** Rute Loureiro, Silvia Magalhães-Novais, Katia A. Mesquita, Ines Baldeiras, Isabel S. Sousa, Ludgero C. Tavares, Ines A. Barbosa, Paulo J. Oliveira, Ignacio Vega-Naredo

**Affiliations:** ^1^ CNC-Center for Neuroscience and Cell Biology, University of Coimbra, Coimbra, Portugal; ^2^ Department of Life Sciences, University of Coimbra, Coimbra, Portugal; ^3^ School of Medicine, University of Coimbra, Coimbra, Portugal; ^4^ Department of Morphology and Cell Biology, University of Oviedo, Oviedo, Spain

**Keywords:** melatonin, mitochondria, metabolism, dichloroacetate, cancer stem cells

## Abstract

Although melatonin oncostatic and cytotoxic effects have been described in different types of cancer cells, the specific mechanisms leading to its antitumoral effects and their metabolic context specificity are still not completely understood. Here, we evaluated the effects of melatonin in P19 embryonal carcinoma stem cells (CSCs) and in their differentiated counterparts, cultured in either high glucose medium or in a galactose (glucose-free) medium which leads to glycolytic suppression and increased mitochondrial metabolism. We found that highly glycolytic P19 CSCs were less susceptible to melatonin antitumoral effects while cell populations relying on oxidative metabolism for ATP production were more affected. The observed antiproliferative action of melatonin was associated with an arrest at S-phase, decreased oxygen consumption, down-regulation of BCL-2 expression and an increase in oxidative stress culminating with caspase-3-independent cell death. Interestingly, the combined treatment of melatonin and dichloroacetate had a synergistic effect in cells grown in the galactose medium and resulted in an inhibitory effect in the highly resistant P19 CSCs. Melatonin appears to exert its antiproliferative activity in P19 carcinoma cells through a mitochondrially-mediated action which in turn allows the amplification of the effects of dichloroacetate, even in cells with a more glycolytic phenotype.

## INTRODUCTION

Melatonin (N-acetyl-5-methoxytryptamine), the main pineal hormone that relays light/dark cycle information to the circadian system, can also be produced in other tissues [1]. Melatonin amphiphilic characteristics allow it to reach any cell, compartment or body fluid [2]. Besides its well-known functions in circadian and seasonal rhythms, melatonin and its metabolites also decrease oxidative stress by acting both as direct free radical scavenger and by stimulating the activity and expression of antioxidant enzymes [3–6]. Additionally, several other activities have been attributed to melatonin: the regulation of the immune system [7], the modulation of mitochondrial activity [8], as well as regulation of cell death [9] and autophagy [10], and an intrinsic antitumoral activity [11]. Due to the multitude of its actions, a role in maintaining healthy aging has been ascribed to melatonin [12].

Two models have been proposed to explain tumor heterogeneity: the stochastic model that assumes that all cancerous cells have the ability to proliferate and regenerate a tumor [13] and the cancer stem cell model that hypothesizes that similarly to normal tissues, tumors are composed of a mixed population of cells at varying states of differentiation. These differentiated cancer cells (dCCs) are typically unable to initiate a tumor and normally derive from stem-like counterparts with the ability to proliferate indefinitely. Recently, a new cancer stem cell hypothesis suggests that cancer stem cells could be mostly originated from stochastic effects during DNA replication in normal adult stem cells [14]. Regardless of the true mechanism, cancer stem cells are considered the driving force behind cancer development and progression. It is believed that cancer stem cell differentiation leads to the production of multiple lineages ultimately forming the tumor bulk [15]. In this regard, cancer stem cells have emerged as an important chemotherapeutic target, as their ability to evade treatments provides a likely explanation for tumor re-growth. Although cancer stem cells were only initially described in 1997 for acute myeloid leukemia [16], they have been further identified and isolated from bone marrow, brain, breast, pancreas, skin, neck, nervous system, colon and prostate cancers [17, 18]. Approaches to eliminate cancer stem cells and avoid tumor re-growth include the depletion of tumor blood supply, differentiation therapies, developmental signaling pathways, DNA checkpoint proteins, and modulation of the cellular redox state [18–20].

P19 embryonal carcinoma stem cells (CSCs) constitute an appropriate model for the study of cancer stem cell maintenance and differentiation. In fact, upon its loss of pluripotency through differentiation with retinoic acid these cells retain their immortalized phenotype [21, 22]. We had previously observed that P19 CSCs possess specific mitochondrial and metabolic properties that are altered during cell differentiation. These properties are inter-connected with pluripotency and resistance to the mitochondrial agent dichloroacetate. Thus, the stimulation of mitochondrial activity by culturing P19 stem cells in galactose (glucose-free), glutamine/pyruvate- containing medium reduced their glycolytic phenotype and stemness, triggered cell differentiation, and increased the susceptibility of P19 cells to dichloroacetate [23].

Although sufficient evidence supports the antitumoral function of melatonin in some tumor types [24], little is known about the functional effects of this indolamine in cancer cells expressing a stem cell-like phenotype and, particularly, how that effect depends on mitochondrial activity. In general, factors which allow melatonin to recognize context specificity and induce apoptosis in some types of cancer cells only are not completely known [24]. In the present study, we analyze the effect of melatonin in the P19 CSCs model which allows testing the effect of different molecules in distinct metabolic contexts and stemness stages. Interestingly, we found that only cells relying on a more oxidative metabolism for ATP production were susceptible to melatonin. Thus, this work aims the understanding of the mechanisms that make these cells vulnerable to melatonin in comparison with their glycolytic counterparts.

## RESULTS

The P19 cell model allows testing the effects of melatonin on the same cancer cell line within different degrees of pluripotency, differentiation and mitochondrial activity [23].

### Melatonin decreased P19 cell mass only when oxidative metabolism was used for ATP production

**Figure 1:**
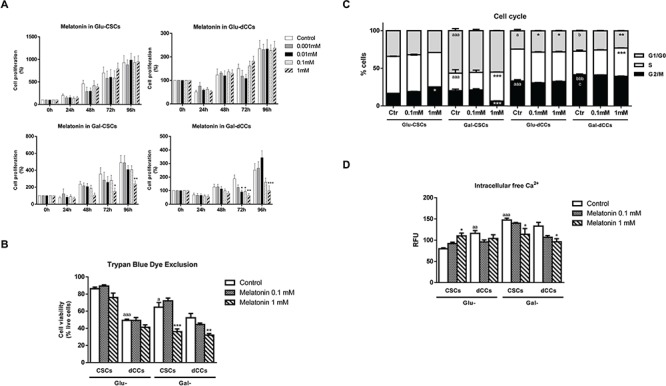
Melatonin showed antiproliferative effects only in P19 cells which were reliable in oxidative metabolism for ATP production Melatonin effects on cell viability were measured in the four types of P19 cells: stem (CSCs) and differentiated (dCCs) lines, growing in glucose (Glu) and in modified galactose (Gal)-containing media. **A.** The sulforhodamine B (SRB) assay shows a cytotoxic effect of 1mM melatonin in Gal-CSCs and Gal-dCCs. Data represent the average percentage of SRB absorbance with respective time 0 ± SEM from at least three independent experiments. *p < 0.05; **p < 0.01; ***p < 0.001 vs. control. **B.** Cell viability determined by trypan blue dye exclusion assay after 72 hours of treatment with melatonin confirms the resistance of P19 cells cultured in high glucose medium. Data are expressed as percentage of live cells ± SEM from at least three independent experiments. * vs. control; a vs. Glu-CSCs. **C.** Cell cycle was analyzed by flow cytometry using propidium iodide in the four types of P19 cancer cells, untreated (Ctr) and treated with melatonin (0.1 and 1 mM) during 72 hours. Data are expressed as percentage of cells in G1/G0, S and G2/M ± SEM from three independent experiments. **D.** Intracellular levels of free calcium were detected by Fluo-4 fluorescence. Data are means ± SEM from at least three separate experiments. Statistical comparisons: * vs. Ctr; a vs. Glu-CSCs; b vs. Gal-CSCs; c vs. Glu-dCCs. The number of symbols marks the level of statistical significance: one for *p* < 0.05, two for *p* < 0.01, and three for *p* < 0.001.

To assess the effect of different concentrations of melatonin (0.001, 0.01, 0.1 and 1 mM) on P19 four stages of differentiation (Glu-CSCs, Glu-dCCs, Gal-CSCs and Gal-dCCs), cell mass was evaluated after 24, 48, 72 and 96 hours of treatment. The decreasing effect of melatonin on cell mass was dependent on the medium used for cell growth. At 72 hours of incubation, 1 mM melatonin significantly decreased Gal-CSCs and Gal-dCCs cell mass, which was more evident in Gal-dCCs (Figure 1A). In fact, the concentration required for half maximal inhibitory effect was considerably lower in Gal-dCCs than in Gal-CSCs (Table 1).

**Table 1:** Computing simulation for obtaining the half maximal inhibitory concentration and the combination index in P19 cells treated with dichloroacetate (DCA) and melatonin (MEL)

	IC_50_(mM)		CI	
	MEL	DCA	10 mM DCA + 0.1 mM MEL	10 mM DCA + 1 mM MEL
Glu-CSCs	n/a	52.915	n/a	n/a
Glu-dCCs	n/a	9.955	n/a	n/a
Gal-CSCs	1.930	19.988	0.954	0.273
Gal-dCCa	0.024	14.360	2.052	0.496

Dose-response values IC_50_ (dose required for median effect) and the combination index (CI) were obtained with CompuSyn software for drug combinations and for general dose effect analysis, using data from cell mass studies in P19 embryonal carcinoma stem cells (CSCs) and differentiated cancer cells (dCCs) grown in glucose media (Glu) and in glucose-free galactose-containing media (Gal). The CI value reflects the extent of synergy or antagonism: CI < 0.9, synergy; 0.9 < CI < 1.1, additive effect; CI > 1.1, antagonism. n/a: not applicable.

The trypan blue dye exclusion assay was carried out to determine the effects on cell viability after 72 hours of treatment with 0.1 and 1 mM melatonin (Figure 1B). Hence, only cells cultured in galactose (glucose-free), glutamine/pyruvate- containing media which relied more in oxidative metabolism for ATP production were susceptible to 1 mM melatonin (*p* < 0.01). Considering these observations, one can ask what makes these cells more susceptible to melatonin in comparison to their high glucose medium counterparts.

### Melatonin reduced intracellular calcium concentration and induced S-phase arrest in P19 cells grown in the modified galactose-containing media

In order to verify whether the effect of melatonin was mediated by any alteration on cell cycle progression, flow cytometry analysis with propidium iodide was performed in the four groups of P19 cells treated with melatonin (0.1 and 1 mM) during 72 hours. As expected, all differentiated P19 cell groups generated by either the addition of retinoic acid (Glu-dCCs, Gal-dCCs) or by culture in the modified galactose-containing medium (Gal-CSCs), presented differences regarding cell cycle progression when compared to the undifferentiated group. Thereby, Gal-CSCs significantly increased the percentage of cells in G1/G0 phase at expenses of reducing cells at S-phase (*p* < 0.001 vs. Glu-CSCs). Moreover, P19 Glu-dCCs presented an arrest on G2/M phase (*p* < 0.001) when compared to their stem counterpart (Glu-CSCs). Similarly, P19 Gal-dCCs prolonged its G2/M phase at the expense of a reduction on G1/G0 phase (*p* < 0.05) when compared to Gal-CSCs. Therefore, when compared to the groups previously shown to be more resistant to melatonin (P19 cells grown on high glucose medium), all other groups of P19 cells showed a significant decrease in S-phase after treatment with melatonin.

The effect of melatonin on cell cycle progression was dependent on the metabolic and differentiation status of the cells. In this regard, 1 mM melatonin 72 hours treatment induced an arrest at G2/M and G1/G0 phases respectively for the resistant Glu-CSCs and Glu-dCCs groups (*p* < 0.05). On the other hand, 1 mM melatonin induced an arrest at S-phase in both P19 cell groups cultured in galactose (glucose-free), glutamine/pyruvate- containing medium (*p* < 0.001) at expenses of reducing the number of cells on G2/M phase for Gal-CSCs, and on G1/G0 phase for Gal-dCCs (Figure 1C).

Melatonin modulates calcium homeostasis [25], a critical step to maintain a regular cell cycle progression. The four groups of P19 cells showed different basal levels of intracellular free calcium, being the highest concentration observed in P19 cells grown in galactose (glucose-free), glutamine/pyruvate- containing medium. In these groups of P19 cells cultured in the modified galactose media, 1 mM melatonin along 72 hours treatment resulted in decreased amount of free calcium (*p* < 0.05) in clear contrast to the results in the resistant Glu-CSCs (Figure 1D).

### Melatonin altered mitochondrial membrane potential, oxygen consumption and ATP content in P19 cells

Considering that the antiproliferative action of melatonin was only observed in P19 cells with active mitochondrial metabolism, we propose that this effect may be mediated through a direct interaction with the referred organelle.

**Figure 2:**
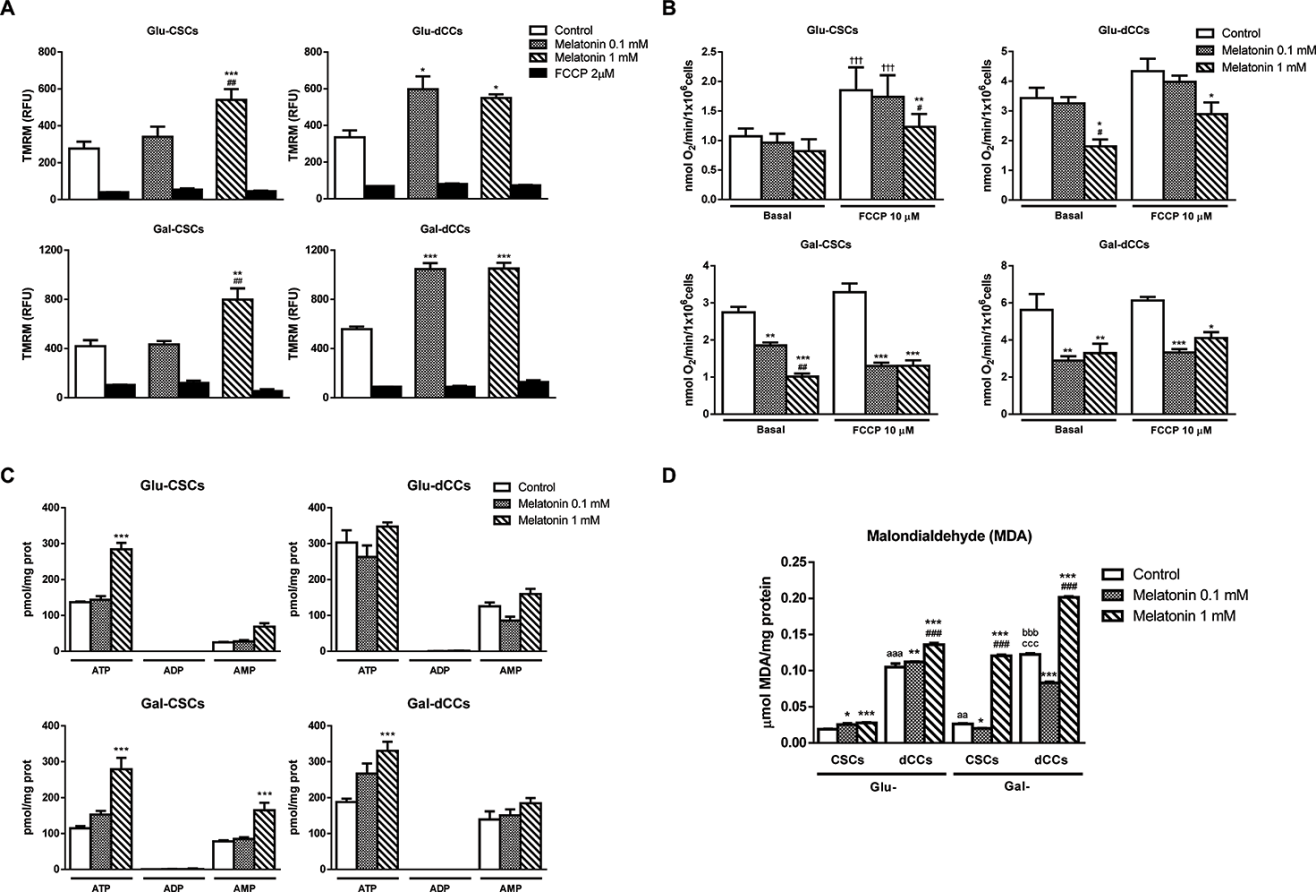
Melatonin altered mitochondrial membrane potential, oxygen consumption and ATP production in P19 cells **A.** Accumulation of the potentiometric dye tetramethylrhodamine methyl ester (TMRM) was measured by flow cytometry in all experimental groups: stem (CSCs) and differentiated (dCCs) P19 cells, growing in glucose (Glu) and in modified galactose (Gal)-containing media, and treated with 0.1 and 1 mM melatonin during 72 hours. Data are shown as relative fluorescence units (RFU) that represent the mean average of geometric mean values ± SEM from at least three independent experiments. Carbonyl cyanide 4-(trifluoromethoxy)phenylhydrazone (FCCP) (2 μM) was used as negative control. Statistical comparisons: * vs. control; # vs. 0.1 mM melatonin. **B.** Oxygen consumption displayed as nmol of O_2_ per minute and per 10^6^ cells was decreased after the treatment with melatonin in Glu-dCCs, Gal-CSCs and Gal-dCCs. Ten μM FCCP was added to test maximal respiration. Data are means ± SEM from at least three independent experiments. Statistical comparisons: * vs. control; # vs. 0.1 mM melatonin; † vs. basal. **C.** ATP, ADP and AMP measurements obtained by high-performance liquid cromatography and expressed as pmol per mg of protein indicated an increased ATP production after 72 hours of treatment with 1 mM melatonin, particularly in cells cultured in galactose medium. Data are means ± SEM from *n* = 6 experiments. **D.** Measurement of malondialdehyde (MDA) by high-performance liquid cromatography in the four groups of P19 cells revealed an increased lipid peroxidation after 72 hours of treatment with 1 mM melatonin, especially in cells cultured in modified galactose medium. Data are means ± SEM for *n* = 6 experiments. Statistical comparisons: * vs. control; # vs. 0.1 mM melatonin; a vs. Glu-CSCs; b vs. Gal-CSCs; c vs. Glu-dCCs. The number of symbols marks the level of statistical significance: one for *p* < 0.05, two for *p* < 0.01, and three for *p* < 0.001.

In all P19 cell groups, melatonin increased mitochondrial membrane potential, reaching significant values with 1 mM melatonin for both groups of CSCs (Glu-CSCs and Gal-CSCs) and with 0.1 mM melatonin for both dCCs groups (Figure 2A). Since the mitochondrion couples the maintenance of mitochondrial membrane potential with electron transport in the respiratory chain and with ATP synthesis, we next measured mitochondrial respiration. Figure 2B shows no effects on basal oxygen consumption in glycolytic Glu-CSCs treated with melatonin. In contrast, melatonin decreased basal respiration of more oxidative cells (Glu-dCCs, Gal-CSCs, Gal-dCCs), which was especially relevant for cells grown in galactose medium (*p* < 0.01). Interestingly, 10 μM FCCP increased oxygen consumption only in Glu-CSCs (*p* < 0.001) and did not increase oxygen consumption up to a similar maximal capacity in melatonin-treated groups which were shown to have decreased basal respiration levels. Furthermore, although basal oxygen consumption was not affected by melatonin in Glu-CSCs, the addition of FCCP to cells treated with 1 mM melatonin did not result in increased respiration, suggesting that melatonin impairs respiration even in the high glycolytic and resistant Glu-CSCs. Overall, these results point out for a direct action of melatonin on the mitochondrial electron transport chain in all groups of P19 cells, especially in those with an active oxidative metabolism. Conversely, its effects on the mitochondrial transmembrane electric potential suggest another target. Intriguingly, 1 mM melatonin significantly increased ATP content in Glu-CSCs, Gal-CSCs and Gal-dCCs (Figure 2C). Despite this, both ADP and AMP levels, as well as energy charge and the percentage of ATP in the total adenine nucleotide pool remained unchanged after treatment with melatonin in the four groups of P19 cells.

### Melatonin showed a pro-oxidant effect, reduced BCL-2 expression and induced a caspase-3-independent cell death in P19 cells with oxidative metabolism

The disruption of the mitochondrial electron transport chain results in a higher reactive oxygen species (ROS) production [26]. Differentiated P19 cells (Glu-dCCs and Gal-dCCs) presented higher malondialdehyde (MDA) content, a classical marker of oxidative stress, when compared to their stem counterparts (*p* < 0.001). Similarly, cells cultured in galactose (glucose-free), glutamine/pyruvate- containing media (Gal-CSCs and Gal-dCCs) also presented higher MDA content than their high glucose-cultured counterparts. Despite these basal differences, 1 mM melatonin significantly increased MDA levels in all P19 cell groups compared to their corresponding controls (*p* < 0.001), especially in cells cultured in galactose medium (Figure 2D). This effect may be related with the ability of melatonin to decrease the proliferation rate of highly oxidative cells. Intriguingly, although 0.1 mM melatonin also impacted oxygen consumption of cells cultured in galactose media, this concentration also exhibited an antioxidant effect.

**Figure 3:**
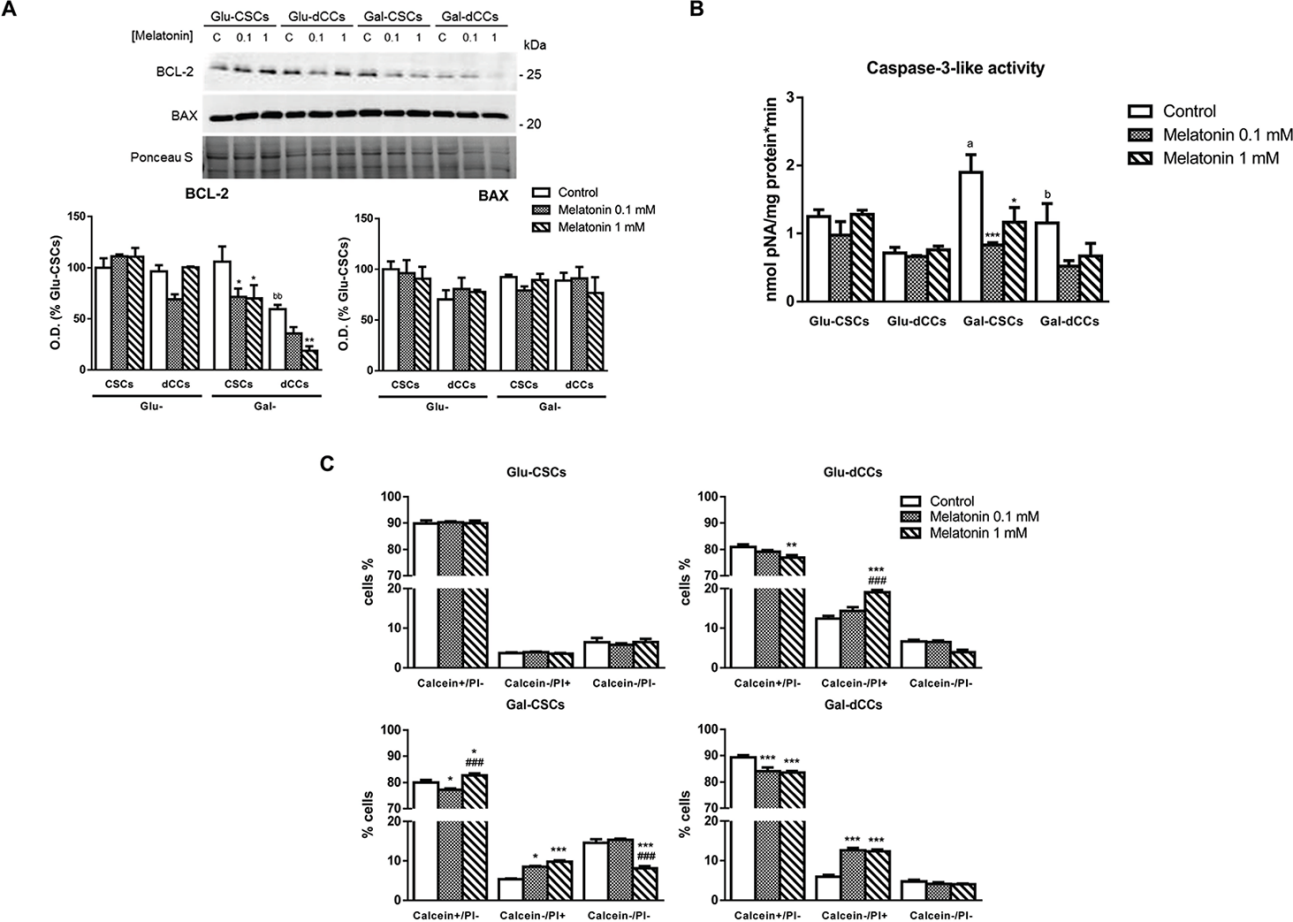
Melatonin induced caspase-3-independent cell death in P19 cells presenting oxidative metabolism **A.** Protein levels of BCL-2 and BAX revealed no changes in BAX content but evident alterations in BCL-2 especially after treating P19 cells cultured in galactose with melatonin. Treatments: control (C), melatonin 0.1 mM (0.1), and melatonin 1 mM (1). Bar charts show means of optical density (O.D.) ± SEM expressed as percentage of Glu-CSCs, from at least three separate immunoblots. **B.** Caspase-3-like activity determined in the four types of P19 cells: P19 stem (CSCs) and differentiated (dCCs) cells, grown in glucose (Glu) and modified galactose media (Gal), using DEVD linked to p-nitroaniline (pNA) was not induced by the treatment with melatonin during 72 hours. Data are expressed as means (nmol pNA per minute and mg protein) ± SEM from at least three independent experiments. **C.** However, live/dead assay with calcein-AM and propidium iodide (PI) showed a higher percentage of dead cells (calcein-/PI+) in melatonin-treated cells which present a higher oxidative metabolism (Glu-dCCs, Gal-CSCs and Gal-dCCs). Data are expressed as percentage of viable cells (calcein+/PI-), dead cells (calcein-/PI+), and cell debris (calcein-/PI-) ± SEM from at least three independent experiments. Statistical comparisons: * vs. control; # vs. 0.1 mM melatonin; a vs. Glu-CSCs; b vs. Gal-CSCs. The number of symbols marks the level of statistical significance: one for *p* < 0.05, two for *p* < 0.01, and three for *p* < 0.001.

Since excessive ROS production by mitochondria plays a preponderant role in mitochondrial outer membrane permeabilization, we investigated alterations in BCL-2 and BAX protein content. We found no changes in BAX content in whole-cell extracts in any of the analyzed cell groups. However, melatonin-treated cells cultured in galactose media showed a decreased content of the antiapoptotic protein BCL-2, while this effect was not found when cells were grown in high glucose media (Figure 3A). The observed decrease of BCL-2 content in melatonin-treated and galactose-cultured cells suggests that the intrinsic apoptotic pathway may have been activated. However, our data did not show the expected increase in caspase-3-like activity (Figure 3B). Interestingly, untreated Gal-CSCs show the highest activity of caspase-3 when compared to Glu-CSCs (*p* < 0.001). This observation may occur as consequence of the forced metabolic remodeling and its associated differentiation process induced by the galactose (glucose-free), glutamine/pyruvate- containing medium. Nonetheless, the calcein-AM and propidium iodide Live/Dead assay confirmed that 1 mM melatonin increases the percentage of dead cells (calcein-/propidium iodide+) in cell populations with higher mitochondrial metabolism (Gal-CSCs and Gal-dCCs; *p* < 0.001). Moreover, this effect was also detected in Glu-dCCs (*p* < 0.001) where a tendency for decreased viability was measured by the trypan blue assay. Finally, 0.1 mM melatonin also increased the percentage of dead cells but only in cells grown in galactose (glucose-free), glutamine/pyruvate- containing medium (Figure 3C).

### Dichloroacetate enhanced the antiproliferative effect of melatonin

**Figure 4:**
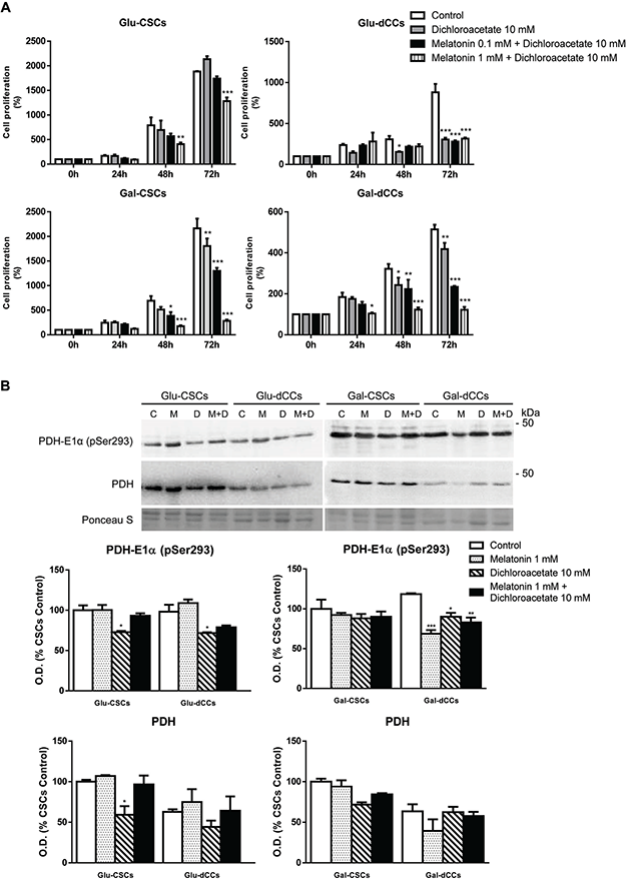
Effect of the combined treatment with melatonin and dichloroacetate (DCA) in P19 embryonal carcinoma stem (CSCs) and differentiated (dCCs) cells, grown in glucose (Glu) and galactose (Gal) media **A.** The sulforhodamine B (SRB) assay shows a decrease in cell mass after 72 hours of the combined treatment of the Glu-CSC group with 1 mM melatonin and 10 mM dichloroacetate. Data represent the average percentage of SRB absorbance with respective time 0 ± SEM from at least four independent experiments. **B.** Content of pyruvate dehydrogenase (PDH) and PDH-subunit E1α phosphorylated at Ser293 site in the four types of P19 cells studied reveals that melatonin was only able to contribute to dichloroacetate effect by reducing PDH phosphorylation in Gal-dCCs. Treatments: control (C), melatonin 1 mM (M), dichloroacetate 10 mM (D), and melatonin 1 mM + dichloroacetate 10 mM (M+D). Ponceau S was used to normalize sample loading. Bar charts show means of normalized optical density (O.D.) expressed as percentage of control ± SEM from three separate immunoblots. *p < 0.05; **p < 0.01; ***p < 0.001 vs. control.

The above results suggest that melatonin antitumor effects are dependent on cellular oxidative metabolism and are caspase-3 activity-independent. Therefore, Glu-CSCs presented a resistant profile which might be related to their predominantly glycolytic metabolism. As melatonin appears to affect only cells relying on mitochondrial metabolism, we next questioned whether a combined treatment with dichloroacetate could affect Glu-CSCs. As shown in Figure 4A, co-treatment with 10 mM dichloroacetate and 1 mM melatonin resulted in decreased Glu-CSCs cell mass (*p* < 0.01 and *p* < 0.001 for 48 and 72 hours of treatment, respectively). Although the calculated combination index with 10 mM dichloroacetate and 0.1 mM melatonin suggested additive and antagonistic effects in Gal-CSCs and Gal-dCCs respectively, the combination of 10 mM dichloroacetate with 1 mM melatonin clearly exerted a synergistic effect in both groups of galactose media-grown cells (Table 1). As dichloroacetate activates pyruvate dehydrogenase (PDH) by inhibiting pyruvate dehydrogenase kinase (PDHK), we evaluated phospho(Ser293)-PDH after 72 hours treatment with 1 mM melatonin and 10 mM dichloroacetate. However, treatment with dichloroacetate alone was able to significantly reduce phospho(Ser293)-PDH in Glu-CSCs, Glu-dCCs and Gal-dCCs. Furthermore, treatment with melatonin, alone or in combination with dichloroacetate, did not alter the phosphorylation status of PHD in all types of P19 cells with the exception of Gal-dCCs where a significant decrease of phospho(Ser293)-PDH (*p* < 0.05) was observed (Figure 4B). Although our results suggest that this pathway might not be directly involved in melatonin effects, the result from the co-treatment in Glu-CSCs and the decrease in phospho(Ser293)-PDH observed in melatonin-treated Gal-dCCs revealed significant information about the possible contribution of this pathway.

**Figure 5:**
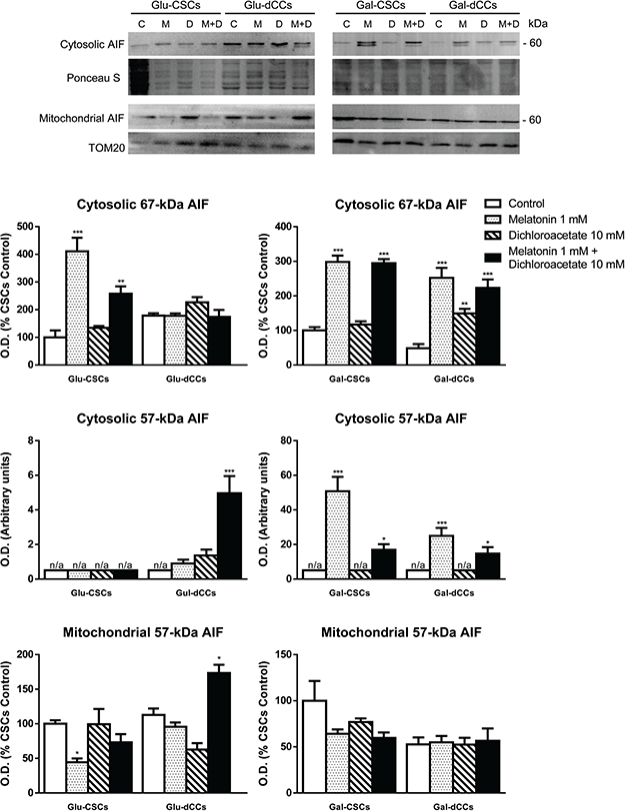
Representative immunoblot images for apoptosis-inducing factor (AIF) in cytosolic and mitochondrial extracts from P19 stem (CSCs) and differentiated (dCCs) cells, grown in glucose (Glu) and galactose (Gal) media, show a higher cytosolic localization of ~57 kDa form of AIF in cells grown in galactose media, treated with melatonin, alone or in combination with dichloroacetate Treatments: control (C), melatonin 1 mM (M), dichloroacetate 10 mM (D), and melatonin 1 mM + dichloroacetate 10 mM (M+D). Ponceau S and TOM20 were used to normalize sample loading in cytosolic and mitochondrial extracts, respectively. Bar charts show means of normalized optical density (O.D.) ± SEM, from at least three separate immunoblots. Statistical comparisons: *p < 0.05; **p < 0.01; ***p < 0.001 vs. control. n/a: no band detected (O.D. = 0).

To determine whether apoptosis-inducing factor (AIF) release from mitochondria is involved in the antiproliferative effects elicited by melatonin, and possibly confirm caspase-independent cell death, we measured AIF content in mitochondrial and cytosolic fractions. The results showed a higher cytosolic 67-kDa AIF content in Glu-CSCs and in both types of galactose media-grown P19 cells either treated with melatonin alone or in combination with dichloroacetate (*p* < 0.05). Besides the observed increase in cytosolic 67-kDa AIF content, an additional cytosolic band of ~57 kDa was identified in melatonin-treated cells grown in galactose medium (Gal-CSCs and Gal-dCCs) and in Glu-dCCs treated with both melatonin and dichloroacetate. This additional band is likely to correspond to a form of AIF which translocates to the nucleus where it triggers caspase-3-independent type of cell death [27] (Figure 5).

## DISCUSSION

Several metabolic properties distinguish cancer cells from normal healthy cells [28], including a decreased mitochondrial ATP production under normoxia, a phenomenon termed Warburg effect [29–31]. Similarly, normal stem cells rely on glycolysis for ATP production. In fact, a recent report suggests that cancer initiating cells may be mostly derived from normal adult stem cells [14]. However, the exact mechanisms by which cancer cells maintain an anaerobic metabolism in the presence of oxygen and the relationship between carcinogenesis and stem cell metabolism are not completely understood [32]. We have previously documented the different metabolic signatures of P19 CSCs and dCCs. P19 CSCs are highly glycolytic and their differentiation is characterized by a more oxidative metabolism marked by a noticeable mitochondrial remodeling. Although P19 CSCs and dCCs have similar amounts of mitochondria, these organelles undergo profound changes in their morphology, biogenesis and physiology along the differentiation process, culminating with the establishment of a long filamentous, polarized and active network [23]. These alterations are accompanied by reduction in stemness and cell growth suggesting a link between pluripotency, proliferation and mitochondrial metabolism. In fact, when forcing mitochondrial metabolism by growing P19 cells in galactose (glucose-free), glutamine/pyruvate- containing medium (Gal-CSCs), cells increase their mitochondrial activity, reduce proliferation and pluripotency and, spontaneously, differentiate. In addition, when P19 Gal-CSCs are treated with retinoic acid, the resultant cells (Gal-dCCs) show the highest degree of mitochondrial development and function [23].

The glycolytic metabolism observed in P19 CSCs is also linked with a drug-resistant phenotype. Thus, P19 CSCs were shown to be resistant to dichloroacetate, a mitochondrial drug that reverses the abnormal metabolism of cancer cells by shifting it from glycolysis to glucose oxidation [23]. This mitochondria-mediated cell death suppression seems to be a common feature among different types of CSCs [33].

As the mechanism by which melatonin is cytotoxic towards some cancer cells is still uncertain, the present work investigates the impact of cell differentiation and metabolism on melatonin action. This was achieved by using P19 cells at four different differentiation stages: Glu-CSCs, Glu-dCCs, Gal-CSCs, and Gal-dCCs. The results obtained here revealed that melatonin did not affect high glucose-grown CSCs. In fact, only cells which rely more in oxidative phosphorylation for ATP synthesis, particularly those cells grown in galactose medium were susceptible to the highest concentration of melatonin (1 mM). In addition, inhibition of proliferation by melatonin was also observed in Gal-dCCs at lower concentrations. As stated above, Gal-dCCs are the cell group with the largest degree of cell differentiation, morphological heterogeneity (Supplementary Figure S1) and up-regulated mitochondrial function, which make these cells more susceptible to antiproliferative agents. In agreement, we have previously demonstrated that Gal-dCCs present a more open conformation of the mitochondrial permeability transition pore that is not able to respond to cyclosporin A [23]. Resistance to drug therapy constitutes an important hallmark of CSCs, which seems to be strongly related with an altered cell metabolic profile. Accordingly, some tumor cells are unable to produce ATP in a mitochondria-dependent manner resulting in a mitochondrial-apoptosis resistant phenotype [28, 33] that is also characteristic of many stem cells [34]. Our present results suggest that melatonin-dependent antiproliferative effect requires an active mitochondrial metabolism. Therefore, if melatonin acts throughout a mitochondria-mediated manner, lower concentrations of melatonin would be expected to be more efficient in revealing its antiproliferative effects in Gal-dCCs than in other types of P19 cells expressing a more resistant and undifferentiated phenotype. It is known that the cytostatic or cytotoxic effects of melatonin in tumor cells vary depending on the cell type and its concentration, being the antiapoptotic actions of melatonin typically described at millimolar concentrations only in specific cancer types [11]. Consequently, our findings highlight the importance of mitochondrial and cell differentiation therapies when using melatonin against cancer cells with more undifferentiated stem cell-like signatures.

Embryonic stem cells (ESCs) exhibit a special cell cycle structure that plays a role in stem cell maintenance [35], being characterized by short G1 and G2 phases and by a high proportion of cells in S-phase. In fact, the stem cell cycle plays a role in the regulation of cell fate decisions [36]. We found that cell cycle is remodeled to the canonical cycle with prolonged G1 phase when P19 cells are forced to rely on mitochondrial metabolism for ATP production. Under these conditions, melatonin had a higher effect and induced an arrest at S-phase. However, this arrest at S-phase induced by high doses of melatonin was not equivalent in Gal-CSCs and Gal-dCCs since it was produced at the expenses of reducing the number of Gal-CSCs cells on G2/M phase and Gal-dCCs cells on G1/G0 phase. These differences may come as consequence of the heterogeneity within cell populations. Both types of cells, Gal-CSCs and Gal-dCCs, are composed of a range of different cell populations with different cell cycle regulation, morphology, growth rate, differentiation patterns and metabolism [23] that may lead to divergent responses upon melatonin treatment. In glycolytic and resistant cells, melatonin induced alternative types of cell cycle arrest, namely at phases G2/M and G1/G0 in Glu-CSCs and Glu-dCCs respectively. Recent studies reported different results regarding cell cycle progression in cancer cells treated with melatonin. In human colorectal cancer cells, melatonin decreased the S-phase population and induced cell death [37], whereas in osteosarcoma and leukemia cells, melatonin reduced the proportion of cells in the S- and G2/M-phases while increasing cells in G1/G0 phase [38, 39], an effect also observed by us in Glu-dCCs. However, in this work, melatonin-induced cell cycle arrests seem to be incomplete in several cases, probably as a consequence of the observed heterogeneity within cell populations. On the other hand, high concentrations of melatonin increased the number of hepatoma cells in S-phase, showing an antiproliferative effect [40] which was also observed by us in P19 cells cultured in galactose (glucose-free), glutamine/pyruvate- containing medium. Therefore, although melatonin induced alterations in cell cycle progression, those effects depend on the overall metabolic and differentiation state of the cancer cells. Even so, in the more resistant P19 Glu-CSCs, melatonin was able to induce an arrest at G2/M albeit without exerting significant cytostatic effects. Thus, in the P19 CSCs model, melatonin was only able to reduce cell proliferation when the cells lost pluripotency and cell cycle was modified to the canonical structure.

Spontaneous Ca^2+^ oscillations are restricted to the G1/S phase transition in P19 CSCs, suggesting a role for Ca^2+^ in stem cell cycle progression [41]. In fact, we have recently reported that P19 stem cells present low cytosolic calcium levels, which are increased during cell differentiation [23]. The higher concentrations of free calcium found in P19 cells grown in galactose medium are correlated with the changes found on cell cycle progression. Accordingly, the effect of melatonin on free Ca^2+^ was also dissimilar. Glu-CSCs showed increased Ca^2+^ levels upon melatonin treatment while having no effect on cell viability. Conversely, melatonin-treated cells grown in galactose (glucose-free), glutamine/pyruvate- containing medium showed cell cycle arrest at S-phase, decreased cell viability and low intracellular free Ca^2+^ concentrations which, according with our previous results [23], seem to be required at high levels during the process of cell differentiation and mitochondrial maturation. These different results, which depend on the cellular metabolic state, are probably influenced by the lower calcium retention ability of mitochondria from differentiated cells than those from Glu-CSCs [23]. Thus, the effects of melatonin on calcium signaling [25, 42] may play a role in mediating its growth-inhibitory action in cells with an active mitochondrial metabolism.

Melatonin mitigates mitochondrial dysfunction in healthy cells, maintaining membrane potential and optimizing electron transport within the respiratory chain [8, 43, 44]. However, in some melatonin-sensitive cancer cells, mitochondrial depolarization is observed [45, 46]. Although this effect is not observed in other tumor cells [39], it suggests, according to our results, that the disruption of mitochondrial membrane potential is not essential for melatonin-induced antiproliferative actions. Despite this, P19 cells relying on oxidative metabolism showed a decrease in cell respiration after treatment with melatonin, an effect also identified in Glu-CSCs when maximal respiration is induced with FCCP. Although Glu-CSCs present depolarized mitochondria and decreased oxygen consumption, mitochondrial respiration is not completely impaired as cells possess functional OXPHOS machinery but probably decouple respiration from ATP production [23]. Then, when cells undergo differentiation, mitochondria became polarized suggesting a different modulation of mitochondrial potential between stem and differentiated cells. While mitochondrial membrane potential in differentiated cells is maintained by the electron transport chain, in undifferentiated cells this process depends mostly on glycolysis and on ATP hydrolase activity of the F1Fo-ATPase [23]. Our results suggest a direct effect of melatonin in the electron transport chain, although the observed increase of mitochondrial membrane potential may not result from an inhibition of the respiratory chain alone but instead from effects on ATP synthase.

Previous works showed an effect of melatonin in inhibiting the increased respiration resulting from stimulation of Krebs' cycle, protecting mitochondria from oxidative damage [47]. In another study, it was reported that melatonin decreased OXPHOS through complex IV inhibition while increasing the glycolytic efficiency [48], an effect that might explain the resistance of Glu-CSCs to melatonin, as well as the observed decrease in respiration and increased ATP levels. In our previous report, we demonstrated, by blocking OXPHOS with oligomycin, that glycolysis is the primary pathway for ATP production in P19 cells [23]. Although we have found here that 1mM melatonin also produced an increase in the ATP content in Gal-CSCs and Gal-dCCs, we have not found differences regarding the percentage of ATP on the total adenine nucleotide pool between controls and melatonin-treated cells. Thus, the energetic balance was not affected by the treatment with melatonin. It was described that melatonin maintains mitochondrial homeostasis in normal cells since it is able to reduce oxygen consumption while maintaining OXPHOS activity and ATP synthesis [49]. Despite this, the higher amount of adenine nucleotides observed after the treatment with melatonin could also be related with an action of melatonin in triggering and/or directing cell differentiation as previously described [50, 51]. This action would be mediated by its interaction with the retinoic-related orphan receptor alpha (RORA) whose expression is temporally regulated during differentiation of P19 cells into neural lineages [52, 53], as probably occurs in P19 cells grown in galactose that express higher amounts of the neuron-specific marker betaIII-tubulin [23]. In general, our data indicate that the predictable outcome of melatonin treatment depends on the involvement of mitochondrial bioenergetics to cell metabolism.

It is known that melatonin-induced apoptosis in cancer cells is associated with ROS production [45, 54, 55], and that oxidative stress determines cancer cells fate in response to melatonin [11]. Here, we found a link between impaired oxygen consumption, mitochondrial hyperpolarization and oxidative stress generation in cells grown in the modified galactose media and treated with melatonin. Although the precise mechanism by which melatonin induces ROS in cancer cells remains unknown, our results together with data from other authors suggest that ROS produced by the mitochondrial electron transport chain constitutes a key factor in melatonin-induced cell death [39, 55–57] and differentiation [50].

Excessive ROS contributes to mitochondrial outer membrane permeabilization (MOMP), which is mainly controlled by proteins from the BCL-2 family and is an important factor in mediating the intrinsic apoptosis [56]. Previous studies have noted that melatonin alters the balance between BAX and BCL-2 in some cancer cells by up-regulating BAX expression, resulting in MOMP and cytochrome c release [39, 58, 59]. However, in other cancer cells, melatonin induces a decrease in BCL-2 [45, 46, 60]. Here, BAX remained unaltered whereas BCL-2 was down-regulated in the cells grown in galactose media and treated with melatonin, altering the BAX/BCL-2 balance but without causing cytochrome c release (data not shown), probably due to increased mitochondrial membrane potential. Accordingly, we have not detected an increase in caspase-3-like activity. Nonetheless, the Live/Dead assay suggests that the observed decrease in cell proliferation is due to a cytotoxic rather than a cytostatic action of melatonin.

Melatonin increased cytosolic AIF levels in Gal-CSCs and Gal-dCCs, as well as in Glu-CSCs. However, the observed band corresponds to a 67-kDa form of AIF, which is the precursor form containing a mitochondrial-localizing sequence, and which is unable to cause cell death [61]. On the contrary, melatonin seems to exert another undescribed mitochondrial effect by inducing *de novo* synthesis of the AIF precursor protein. After being imported into mitochondria, the mitochondrial localizing sequence contained in 67-kDa AIF is cleaved, resulting in the accumulation of the mature 57-kDa form of the AIF protein. This is described to translocate to the nucleus where it triggers a caspase-3-independent type of cell death [27]. We found a higher cytosolic localization of this ~57 kDa form of AIF in cells cultured in galactose media and treated with melatonin, alone or in combination with dichloroacetate, and in Glu-dCCs treated with melatonin and dichloroacetate.

Nonetheless, the increased percentage of dead cells (calcein -/ PI +) after 72 hours of treatment with melatonin, although moderated, was statistically significant (*p* < 0.001) in cells with a more oxidative metabolism. In these cells, cultured in the galactose medium, more than 50% of the cellular mass was lost after 72 hours of treatment with melatonin. Due to this, the measurement of the percentage of live/dead cells in the remaining population probably represents early (or other) events of cell death as well as processes of selection of the more resistant subpopulations. In fact, we cannot exclude divergent effects of melatonin in the different cell subpopulations. In accordance to this hypothesis, we observed a different pattern of AIF expression in some cells which may indicate the presence of different cell subpopulations with dissimilar susceptibility to activate this pathway (Supplementary Figure S2).

Taking all our results into account, we can infer that melatonin induces a toxic effect in P19 embryonal carcinoma cells via the inhibition of mitochondrial metabolism as described in other types of tumor cells [62]. Thus, P19 Glu-CSCs present a strong resistant phenotype, which seems to be linked to their glycolytic metabolism. In fact, we previously observed that only the P19 cells with an active mitochondrial metabolism are susceptible to the anticancer agent dichloroacetate [23]. Surprisingly, when we combined 1 mM melatonin and 10 mM dichloroacetate, cytotoxicity in the highly resistant Glu-CSCs was observed. These results are of great importance considering that to our knowledge, this is the only treatment showing an efficient and viable effect against P19 Glu-CSCs. Furthermore, the synergistic capability of this treatment combination was observed in P19 cells with the most active mitochondrial metabolism. The mechanism of action of dichloroacetate ultimately concerns the activity of PDH that, according to our previous results, seems to be deregulated and overexpressed in P19 CSCs [23]. However, upon melatonin treatment, no significant changes in the content of PDH on its active (dephosphorylated) form were found, which would allow us to explain its synergistic effect with dichloroacetate. The absence of correlation between the phosphorylation status of PDH and the observed synergistic effect reinforce the hypothesis about a deregulation in the PDK-PDH axis, at least in the more undifferentiated P19 cells. Thus, this deregulation might be related with the preference of primitive cells for a more glycolytic metabolism. In agreement, the group composed of more differentiated and oxidative cells (Gal-dCCs) showed significant changes in phospho(Ser293)-PDH when treated with melatonin and dichloroacetate. Since other mechanisms of PDH regulation may involve other phosphorylation sites [63], our results suggest the contribution of alternative mechanisms which might also be independent of the phosphorylation status of PDH, thus explaining part of the synergistic effect of the combination. For example, it was recently described that dichloroacetate is able to suppress mTOR activity specifically through pyruvate dehydrogenase kinase 4 (PDK4) and independently of PDH [64]. Curiously, it was also described that *Pdk4* gene expression can be increased by melatonin in mice [65].

**Figure 6:**
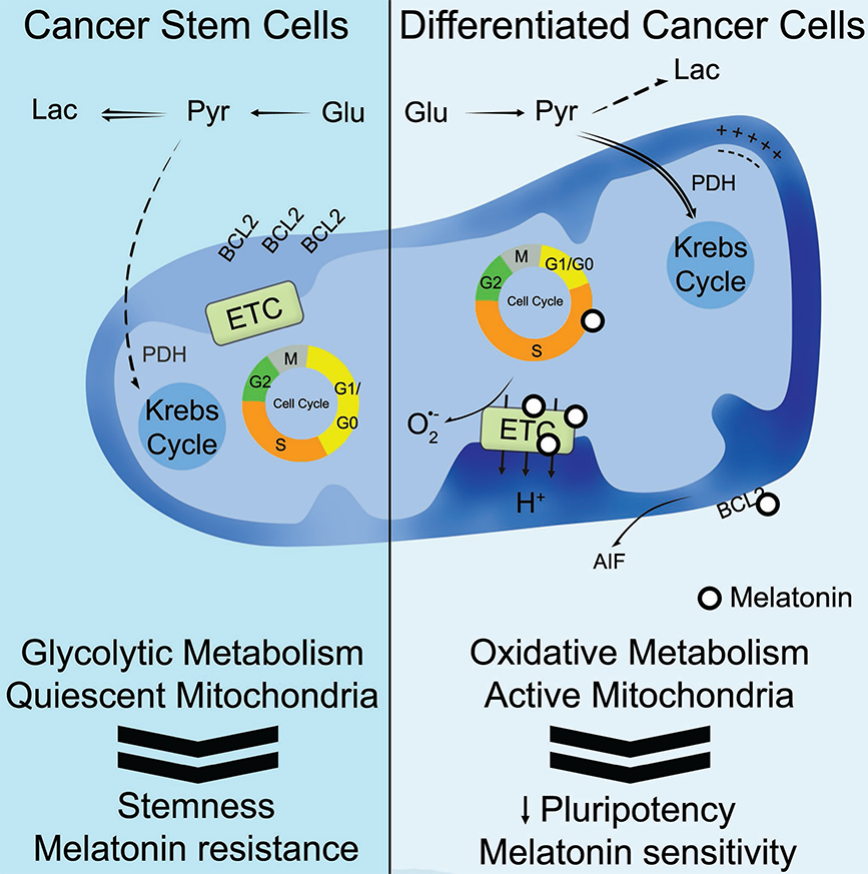
Schematic representation summarizing the proposed role of melatonin in cancer stem cells Our hypothesis ascribes an anti-tumor effect for melatonin only in differentiated cancer cells with an active oxidative metabolism, triggering a type of mitochondrial-mediated cell death which is likely to be characterized by an arrest at S-phase, reduction of the mitochondrial electron transport chain (ETC), generation of reactive oxygen species, BCL-2 down-regulation and AIF releaseThus, the treatment with melatonin and the stimulation of mitochondrial metabolism constitute promising strategies against resistant cancer stem cells.

Nonetheless, the decrease of phospho(Ser293)-PDH observed in Gal-dCCs after treatment with melatonin, reinforces the idea that the anti-tumor actions of melatonin occur at a mitochondrial level. Accordingly, our results suggest that melatonin exerts its anticancer effects in P19 cells with an active oxidative metabolism, triggering a type of mitochondrial cell death which is caspase-3-independent and is probably AIF-mediated (Figure 6). These results are in accordance with previous works developed in other types of cancer cells such as MCF-7 [66].

Mitochondrial activity, function and differentiation strongly impact the antitumoral ability of melatonin which involves, among others, the activation of intrinsic apoptotic pathways, in P19 embryonal carcinoma cells as well as in other types of cancer cells [24]. It is known that AIF controls programmed cells death during early morphogenesis and that its genetic inactivation renders embryonic stem cells resistant to cell death [67]. Consequently, the mechanism of caspase-3-independent cell death, stimulation of mitochondrial differentiation and metabolism, with consequent disruption of the Warburg effect constitute promissory strategies when targeting resistant cancer cells with an embryonic signature.

## MATERIALS AND METHODS

### Cell culture, differentiation and treatment

P19 embryonal carcinoma cells (CRL-1825, ATCC; Manassas, VA, USA) were obtained and cultured in glucose- or galactose (glucose-free)-containing media at 37°C in a 5% CO2 atmosphere. High glucose Dulbecco's modified Eagle's medium (DMEM, D5648; Sigma, St. Quentin Fallavier, France) was supplemented with 10% FBS (10270–106, Gibco; Paisley, UK), 1.8 g/l sodium bicarbonate (S5761, Sigma), 110 mg/l sodium pyruvate (P5280, Sigma) and antibiotic/antimycotic solution (A5955, Sigma). Galactose (glucose-free)-containing medium was prepared using DMEM without glucose (D5030, Sigma) supplemented with 10% FBS, 1.8 g/l sodium bicarbonate, 110 mg/l sodium pyruvate, 1.8 g/l galactose (G5388, Sigma), 0.584 g/l L-glutamine (G3126, Sigma) and antibiotic/antimycotic solution. P19 cancer cells, growing in high glucose DMEM or in galactose (glucose free)-containing DMEM, were maintained in monolayer and passaged every 2–3 days at a 1:20 to 1:30 dilution.

In order to induce cell differentiation, P19 CSCs were seeded at a density of 5.2 × 10^3^ cells/cm^2^ and 1 μM of retinoic acid (R2625, Sigma) was added for 96 hours.

Melatonin (M5250, Sigma) was always freshly dissolved in ethanol and dichloroacetate (347795, Sigma) in water. If not indicated otherwise, twenty-four hours after seeding, P19 CSCs and dCCs grown in both culture media were exposed to 0.1 mM and 1 mM melatonin alone or in combination with 10 mM dichloroacetate (347795, Sigma) during 72 hours. Controls were always treated with an equal amount of vehicle (less than 0.95% ethanol).

### Cell viability/mass

Cell mass was measured by the Sulforhodamine B (SRB) assay as described previously [68]. P19 cells were seeded in in 48-well plates at a concentration of 0.5 × 10^4^ cells/ml for P19 CSCs and 2 × 10^4^ cells/ml for P19 dCCs. Twenty-four hours after seeding, cells were treated with different melatonin concentrations (1 μM, 10 μM, 0.1 mM and 1 mM) alone, or in combination with 10 mM dichloroacetate. Treatments were performed from 24 to 96 hours and samples were collected every 24 hours. After each period of treatment (24, 48, 72 and 96 hours), the medium was removed and wells rinsed with 1% PBS. Cells were then fixed by adding 1% acetic acid in 100% methanol for at least 2 hours at −20°C. Later, the fixation solution was discarded and the plates dried in an oven at 37°C. Next, 250 μl of 0.5% SRB in 1% acetic acid solution were added and incubated at 37°C for 1 hours. The wells were then washed with 1% acetic acid in water and dried. Then, 500 μl of 10 mM Tris, pH 10 were added and the plates shaken for 30 minutes. Finally, 200 μl of each supernatant were transferred in 96-well plates and optical density was measured at 540 nm. The dose-response values IC_50_ (dose required for median effect) and the combination index were calculated using Compusyn software [69, 70].

After selecting the optimal time and concentration of melatonin treatments (0.1 mM, 1 mM melatonin for 72 hours), we performed the trypan blue dye exclusion assay to measure cell viability. Treated and control P19 cells were trypsinized and washed with PBS. 100 μl of cell suspension was aseptically transferred to a 1.5 ml tube and an equal volume of trypan blue (T8154, Sigma) was added for 3 minutes at room temperature. The resuspension was then placed in a dual-use counting slide (145–0011, Bio-Rad; Hercules, CA, USA) and read in a TC20 automated cell counter (145–0102SP, Bio-Rad). Results are shown as percentage of live cells.

### Cell cycle analysis

One million P19 cells were harvested, washed with PBS and fixed by adding 70% cold (–20°C) ethanol. Subsequently, cells were centrifuged at 850 g to remove the ethanol, washed twice with PBS and finally resuspended in 400 μl PBS. 10 μg/ml RNAse (R5000, Sigma) and 20 μg/ml propidium iodide (P4864, Sigma) were added for 30 minutes at 37°C. 20 × 10^3^ events per sample were analyzed by using a FACScalibur flow cytometer (Becton Dickenson, San Jose, CA, USA) with 488 nm excitation and 605 nm emission wavelengths. The percentage of cells in G1/G0, S and G2/M was determined using Modfit LT software (Verity Software House, Topsham, ME, USA).

### Intracellular calcium measurement

Intracellular calcium levels were measured as described in the Fluo-4 Direct Calcium Assay kit (F10472, Invitrogen; Paisley, UK). After seeding 5 × 10^5^ P19 cells in 96 well plates, the medium was removed and 50 μl of pre-warmed HBSS were added. The Fluo-4 Direct calcium reagent was prepared as suggested by the manufacturer and added in a final concentration of 2.5 mM to the plate wells containing HBSS. The plates were incubated for 30 minutes at 37°C and fluorescence (excitation at 494 nm and emission at 516 nm) was measured in a Gemini EM fluorescence multi-plate reader (Molecular Devices, Sunnyvale, CA, USA).

### Mitochondrial transmembrane electric potential

The mitochondrial membrane potential in P19 cells was investigated by measuring tetramethylrodamine methyl ester (TMRM; T668, Invitrogen) fluorescence by flow cytometry (FACScalibur, Becton Dickinson). P19 cells were seeded, differentiated and treated with melatonin at the concentrations described above. After, cells were trypsinized and loaded with 150 nM TMRM for 30 minutes and then evaluated for mean cell fluorescence by flow cytometry. The uncoupler carbonyl cyanide 4-(trifluoromethoxy)phenylhydrazone (FCCP; 2 μM) was used as a control and was added 15 minutes after TMRM loading to cause mitochondrial depolarization.

### Oxygen consumption

Oxygen consumption was measured polarographically with a Clark-type Oxygen electrode (YSI 5331, Yellow Springs Instruments; Yellow Springs, OH, USA) connected to a recorder (BD 121, Kipp&Zonen; Delft, The Netherlands) in a thermostated water-jacketed closed chamber with magnetic stirring. The reactions were performed at 37°C in 1 ml of glucose- or galacose-containing DMEM with 5 × 10^6^ cells. Respiration was sustained with endogenous substrates followed by uncoupling by FCCP (10 μM). Respiration rates were obtained assuming an oxygen concentration of 237 nmol O_2_/ml in the experimental medium at 37°C [71]. In order to confirm the measurement of mitochondrial basal respiration, 1.5 μM rotenone was added to completely inhibit oxygen consumption by inhibiting mitochondrial complex I (NADH ubiquinone oxidoreductase).

### Adenine nucleotide measurement (ATP/ADP/AMP)

ATP, ADP and AMP levels were measured by HPLC. For adenine nucleotide extraction, after one rinse in cold PBS, 0.5 ml PBS and 0.5 ml perchloric acid/EDTA were added to each dish. Cells were scraped from the dishes, placed in 1 ml micro-centrifuge tubes and centrifuged for 2 minutes at 14,000 × g. Pellets were then re-suspended in 50 μl of 1 M NaOH and later used for protein quantification by the BCA protein assay (23227, Thermo Scientific; Rockford, IL, USA). The supernatant was neutralized with 3 M KOH in 1.5 M Tris and centrifuged again. The supernatant was again collected and stored at −80°C until analyzed by reverse-phase high performance liquid chromatography. The chromatographic apparatus was a Beckman-System Gold, consisting of a 126 Binary Pump Model and a 166 Variable UV detector, controlled by computer. The detection wavelength was 254 nm, and the column was a Lichrospher 100RP-18 from Merck (Darmstadt, Germany). An isocratic elution with 100 mM phosphate buffer (KH_2_PO_4_), pH 6.5 and 1% methanol was performed with a flow rate of 1 ml/min. The time required for each analysis was 5 minutes.

### Lipid peroxidation

The content in malondialdehyde (MDA) was measured by high performance liquid chromatography (HPLC) separation. P19 cells were harvested, washed and resuspended in 50 mM phosphate buffer (pH 7.4) and stored at −80°C until used. Lipid peroxidation was assessed by the fluorimetric determination (excitation at 515 nm and emission at 553 nm; FP-2020/2025, Jasco, Tokyo, Japan) of MDA adducts separated by HPLC (Gilson; Middleton, WI, USA) using the ClinRep complete kit (RECIPE; Munich, Germany).

### Western blot analysis

In order to obtain total cellular extracts, P19 cells were harvested by trypsinization, washed with PBS and centrifuged for 5 minutes at 1,000 g. The cellular pellet was resuspended in RIPA buffer (R0278; Sigma) supplemented with 2 mM ditiothreitol (DTT), 100 μM phenylmethylsulfonyl fluoride (PMSF), and a protease inhibitor cocktail (containing 1 μg/ml of leupeptin, antipain, chymostatin, and pepstatin A), physically ruptured by sonication and kept at −80°C until used. Protein contents were determined by using the BCA protein assay (23227; Thermo Scientific). After denaturation at 95°C for 5 minutes in a Laemmli buffer (161–0737; BioRad, Hercules, CA, USA), equivalent amounts of protein (30 μg) were separated by electrophoresis in 12% sodium dodecyl sulfate-polyacrylamide gel electrophoresis (SDS-PAGE) and electrophoretically transferred to a polyvinylidene difluoride (PVDF) membrane. Ponceau S staining was used to ensure equal loading [23]. After blocking membranes with 5% skim milk in TBS-T (50 mM Tris-HCl, pH 8; 154 mM NaCl and 0.1% Tween-20) for 1 hour at room temperature, membranes were incubated overnight at 4°C with the antibodies directed against B-cell lymphoma 2 (BCL-2; 2870), BCL-2-associated X protein (BAX, 2772) and pyruvate dehydrogenase (PDH; 3205) from Cell Signaling (Danvers, MA, USA); and pSer293-PDH-E1α (ab92696) from Abcam (Cambridge, UK), each previously diluted 1:1,000 in blocking buffer (1% skim milk in TBS-T). After three 5 minutes-washes in PBS-T, the membranes were incubated with a dilution (1:10, 000 in blocking buffer) of a corresponding alkaline phosphatase-conjugated secondary antibody (Santa Cruz Biotechnology, Santa Cruz, CA, USA) for 2 hours at room temperature. After three washes in PBS-T for 15 minutes each, membranes were developed with the ECF detection system (RPN5785; GE Healthcare, Piscataway, NJ) and imaged with Versa Doc imaging system (Bio-Rad) according to the manufacturers' protocols. Densities of each band were calculated with Quantity One Software (Bio-Rad) or ImageJ software. All data presented are representative from at least three separate experiments.

### Isolation of mitochondrial and cytosolic extracts

Mitochondrial extracts were isolated harvesting P19 cells by trypsinization and by spinning them down at 1,000 g. Pellets were washed once in cold PBS and centrifuged again (1,000 g) at 4°C. The cell suspension was then resuspended in 0.5 ml of ice cold sucrose buffer (250 mM sucrose, 20 mM K^+^ Hepes pH 7.5, 10 mM KCl, 1.5 mM MgCl_2_, 0.1 mM EDTA, 1 mM EGTA) supplemented right before use with 1 mM DTT, 0.1 mM PMSF and protease inhibitor cocktail containing 1 μg/ml of leupeptin, antipain, chymostatin and pepstatin. The cell suspension was then incubated on ice for 20 to 30 minutes. After incubation, cells were transferred to a pre-cooled tissue homogenizer and homogenized 30 times using a tight pestle, while keeping the homogenizer on ice. Progress was monitored every 20 to 30 strokes under a phase contrast microscope and was stopped when more than 90% of cells were burst.

Homogenized cells were centrifuged at 3,500 g for 5 minutes at 4°C. The supernatant, containing the mitochondrial and cytosolic fractions was collected. Then, the collected supernatant was centrifuged again at 10,000 g during 15 minutes at 4°C. The pellet, corresponding to the mitochondrial fraction was resuspended in 50 μl of sucrose buffer cited above. The supernatant was again centrifuged at 100,000 g during 30 minutes at 4°C. The resulting supernatant contained the cytosolic fraction and was lyophilized in order to concentrate the protein and resuspended in 50 μl of the same sucrose buffer.

Specific proteins in both fractions were semi-quantified by western blotting as described above using antibodies against AIF (sc-13116) from Santa Cruz Biotechnology and cytochrome c (556433) from Becton Dickenson. Densitometry values were normalized to the levels of Ponceau S for cytosolic extracts, and to the content in translocase of the outer membrane 20 (TOM20) (sc-11415; Santa Cruz Biotechnology) for mitochondrial extracts.

### Caspase 3-like activity

P19 cells were trypsinized and washed in PBS. After washing, cells were concentrated by centrifuging at 300 g for 3 minutes and the supernatant was discarded. The pellets were resuspended in a lysis buffer containing 100 mM NaCl, 0.1% CHAPS, 1 mM DTT, 0.1 mM EDTA, 50 mM HEPES pH 7.4. The samples were kept on ice for 20 minutes following by protein quantification through the BCA kit assay (23227; Thermo Scientific). Then, 25 μg of cell protein extracts were aliquoted in an assay buffer (100 mM NaCl, 0.1% CHAPS, 1 mM DTT, 0.1 mM EDTA, 10% glycerol, 50 mM HEPES pH 7.4) supplemented with 100 μM caspase-3 substrate Ac-DEVD-pNA (235400; Merck Millipore, Darmstadt, Germany) and incubated for 2 hours at 37°C. Caspase 3-like activity was accessed following the detection of the chromophore p-nitroaniline (pNA) after cleavage from the labeled substrate Ac-DEVD-pNA. Method calibration was achieved using known concentrations of p-NA.

### Live/dead assay

Calcein-AM (C1430; Invitrogen) and propidium iodide (P4864; Sigma Aldrich) were used to determine the percentages of viable and dead cells. After the treatments with melatonin, 10^6^ P19 cells were harvested, washed, resuspended in HBSS/Ca and loaded with 0.1 μM calcein-AM and 8 μM propidium iodide for 20 minutes at room temperature. Fluorescence was measured by FACS (Becton Dickinson FACScalibur) with 488 nm excitation wavelength. The simultaneous measurement of Calcein/propidium iodide fluorescence was performed using 530/30 nm bandpass filter for Calcein and a 610/20 nm bandpass filter for propidium iodide red fluorescence.

### Statistical analyses

Data are mean values ± SEM and multiple comparisons were carried out using ANOVA followed by the Bonferroni post hoc test. Significance was accepted with *p* < 0.05.

## SUPPLEMENTARY FIGURES



## References

[1] Reiter RJ, Tan DX, Rosales-Corral S, Manchester LC (2013). The universal nature, unequal distribution and antioxidant functions of melatonin and its derivatives. Mini Rev Med Chem.

[2] Hardeland R, Pandi-Perumal SR, Cardinali DP (2006). Melatonin. Int J Biochem Cell Biol.

[3] Galano A, Tan DX, Reiter RJ (2013). On the free radical scavenging activities of melatonin's metabolites, AFMK and AMK. J Pineal Res.

[4] Reiter RJ, Acuna-Castroviejo D, Tan DX, Burkhardt S (2001). Free radical-mediated molecular damage. Mechanisms for the protective actions of melatonin in the central nervous system. Ann N Y Acad Sci.

[5] Tan DX, Hardeland R, Manchester LC, Galano A, Reiter RJ (2014). Cyclic-3-hydroxymelatonin (C3HOM), a potent antioxidant, scavenges free radicals and suppresses oxidative reactions. Curr Med Chem.

[6] Vega-Naredo I, Poeggeler B, Sierra-Sanchez V, Caballero B, Tomas-Zapico C, Alvarez-Garcia O, Tolivia D, Rodriguez-Colunga MJ, Coto-Montes A (2005). Melatonin neutralizes neurotoxicity induced by quinolinic acid in brain tissue culture. J Pineal Res.

[7] Calvo JR, Gonzalez-Yanes C, Maldonado MD (2013). The role of melatonin in the cells of the innate immunity: a review. J Pineal Res.

[8] Acuna-Castroviejo D, Escames G, Rodriguez MI, Lopez LC (2007). Melatonin role in the mitochondrial function. Front Biosci.

[9] Sainz RM, Mayo JC, Rodriguez C, Tan DX, Lopez-Burillo S, Reiter RJ (2003). Melatonin and cell death: differential actions on apoptosis in normal and cancer cells. Cell Mol Life Sci.

[10] Vega-Naredo I, Caballero B, Sierra V, Garcia-Macia M, de Gonzalo-Calvo D, Oliveira PJ, Rodriguez-Colunga MJ, Coto-Montes A (2012). Melatonin modulates autophagy through a redox-mediated action in female Syrian hamster Harderian gland controlling cell types and gland activity. J Pineal Res.

[11] Rodriguez C, Martin V, Herrera F, Garcia-Santos G, Rodriguez-Blanco J, Casado-Zapico S, Sanchez-Sanchez AM, Suarez S, Puente-Moncada N, Anitua MJ, Antolin I (2013). Mechanisms involved in the pro-apoptotic effect of melatonin in cancer cells. Int J Mol Sci.

[12] Hardeland R (2013). Melatonin and the theories of aging: a critical appraisal of melatonin's role in antiaging mechanisms. J Pineal Res.

[13] Campbell LL, Polyak K (2007). Breast tumor heterogeneity: cancer stem cells or clonal evolution?. Cell Cycle.

[14] Tomasetti C, Vogelstein B (2015). Cancer etiology. Variation in cancer risk among tissues can be explained by the number of stem cell divisions. Science.

[15] Reya T, Morrison SJ, Clarke MF, Weissman IL (2001). Stem cells, cancer, and cancer stem cells. Nature.

[16] Bonnet D, Dick JE (1997). Human acute myeloid leukemia is organized as a hierarchy that originates from a primitive hematopoietic cell. Nat Med.

[17] Tai MH, Chang CC, Kiupel M, Webster JD, Olson LK, Trosko JE (2005). Oct4 expression in adult human stem cells: evidence in support of the stem cell theory of carcinogenesis. Carcinogenesis.

[18] Tang C, Ang BT, Pervaiz S (2007). Cancer stem cell: target for anti-cancer therapy. FASEB J.

[19] Jung JW, Park SB, Lee SJ, Seo MS, Trosko JE, Kang KS (2011). Metformin represses self-renewal of the human breast carcinoma stem cells via inhibition of estrogen receptor-mediated OCT4 expression. PLoS One.

[20] Trosko JE (2014). Induction of iPS cells and of cancer stem cells: the stem cell or reprogramming hypothesis of cancer?. Anat Rec (Hoboken).

[21] McBurney MW (1993). P19 embryonal carcinoma cells. Int J Dev Biol.

[22] Mummery CL, Feijen A, Moolenaar WH, van den Brink CE, de Laat SW (1986). Establishment of a differentiated mesodermal line from P19 EC cells expressing functional PDGF and EGF receptors. Exp Cell Res.

[23] Vega-Naredo I, Loureiro R, Mesquita KA, Barbosa IA, Tavares LC, Branco AF, Erickson JR, Holy J, Perkins EL, Carvalho RA, Oliveira PJ (2014). Mitochondrial metabolism directs stemness and differentiation in P19 embryonal carcinoma stem cells. Cell Death Differ.

[24] Bizzarri M, Proietti S, Cucina A, Reiter RJ (2013). Molecular mechanisms of the pro-apoptotic actions of melatonin in cancer: a review. Expert Opin Ther Targets.

[25] Dai J, Inscho EW, Yuan L, Hill SM (2002). Modulation of intracellular calcium and calmodulin by melatonin in MCF-7 human breast cancer cells. J Pineal Res.

[26] St-Pierre J, Buckingham JA, Roebuck SJ, Brand MD (2002). Topology of superoxide production from different sites in the mitochondrial electron transport chain. J Biol Chem.

[27] Otera H, Ohsakaya S, Nagaura Z, Ishihara N, Mihara K (2005). Export of mitochondrial AIF in response to proapoptotic stimuli depends on processing at the intermembrane space. EMBO J.

[28] Barbosa IA, Machado NG, Skildum AJ, Scott PM, Oliveira PJ (2012). Mitochondrial remodeling in cancer metabolism and survival: potential for new therapies. Biochim Biophys Acta.

[29] Chen Z, Lu W, Garcia-Prieto C, Huang P (2007). The Warburg effect and its cancer therapeutic implications. J Bioenerg Biomembr.

[30] Ertel A, Verghese A, Byers SW, Ochs M, Tozeren A (2006). Pathway-specific differences between tumor cell lines and normal and tumor tissue cells. Mol Cancer.

[31] Pedersen PL (2007). Warburg, me and Hexokinase 2: Multiple discoveries of key molecular events underlying one of cancers' most common phenotypes, the “Warburg Effect”, i.e., elevated glycolysis in the presence of oxygen. J Bioenerg Biomembr.

[32] Folmes CD, Dzeja PP, Nelson TJ, Terzic A (2012). Metabolic plasticity in stem cell homeostasis and differentiation. Cell Stem Cell.

[33] Loureiro R, Mesquita KA, Oliveira PJ, Vega-Naredo I (2013). Mitochondria in cancer stem cells: a target for therapy. Recent Pat Endocr Metab Immune Drug Discov.

[34] Shyh-Chang N, Daley GQ, Cantley LC (2013). Stem cell metabolism in tissue development and aging. Development.

[35] White J, Dalton S (2005). Cell cycle control of embryonic stem cells. Stem Cell Rev.

[36] Pauklin S, Vallier L (2013). The cell-cycle state of stem cells determines cell fate propensity. Cell.

[37] Hong Y, Won J, Lee Y, Lee S, Park K, Chang KT (2014). Melatonin treatment induces interplay of apoptosis, autophagy, and senescence in human colorectal cancer cells. J Pineal Res.

[38] Liu L, Xu Y, Reiter RJ (2013). Melatonin inhibits the proliferation of human osteosarcoma cell line MG-63. Bone.

[39] Perdomo J, Cabrera J, Estevez F, Loro J, Reiter RJ, Quintana J (2013). Melatonin induces apoptosis through a caspase-dependent but reactive oxygen species-independent mechanism in human leukemia Molt-3 cells. J Pineal Res.

[40] Ozdemir F, Deniz O, Kaynar K, Arslan M, Kavgaci H, Yildiz B, Aydin F (2009). The effects of melatonin on human hepatoma (Hep G2) cell line. Bratisl Lek Listy.

[41] Resende RR, Adhikari A, da Costa JL, Lorencon E, Ladeira MS, Guatimosim S, Kihara AH, Ladeira LO (2010). Influence of spontaneous calcium events on cell-cycle progression in embryonal carcinoma and adult stem cells. Biochim Biophys Acta.

[42] Benitez-King G, Huerto-Delgadillo L, Anton-Tay F (1991). Melatonin modifies calmodulin cell levels in MDCK and N1E-115 cell lines and inhibits phosphodiesterase activity *in vitro*. Brain Res.

[43] Fischer TW, Zmijewski MA, Wortsman J, Slominski A (2008). Melatonin maintains mitochondrial membrane potential and attenuates activation of initiator (casp-9) and effector caspases (casp-3/casp-7) and PARP in UVR-exposed HaCaT keratinocytes. J Pineal Res.

[44] Leon J, Acuna-Castroviejo D, Escames G, Tan DX, Reiter RJ (2005). Melatonin mitigates mitochondrial malfunction. J Pineal Res.

[45] Bejarano I, Redondo PC, Espino J, Rosado JA, Paredes SD, Barriga C, Reiter RJ, Pariente JA, Rodriguez AB (2009). Melatonin induces mitochondrial-mediated apoptosis in human myeloid HL-60 cells. J Pineal Res.

[46] Rubio S, Estevez F, Cabrera J, Reiter RJ, Loro J, Quintana J (2007). Inhibition of proliferation and induction of apoptosis by melatonin in human myeloid HL-60 cells. J Pineal Res.

[47] Reyes-Toso CF, Rebagliati IR, Ricci CR, Linares LM, Albornoz LE, Cardinali DP, Zaninovich A (2006). Effect of melatonin treatment on oxygen consumption by rat liver mitochondria. Amino Acids.

[48] Sarti P, Magnifico MC, Altieri F, Mastronicola D, Arese M (2013). New Evidence for Cross Talk between Melatonin and Mitochondria Mediated by a Circadian-Compatible Interaction with Nitric Oxide. Int J Mol Sci.

[49] Lopez A, Garcia JA, Escames G, Venegas C, Ortiz F, Lopez LC, Acuna-Castroviejo D (2009). Melatonin protects the mitochondria from oxidative damage reducing oxygen consumption, membrane potential, and superoxide anion production. J Pineal Res.

[50] Luchetti F, Canonico B, Bartolini D, Arcangeletti M, Ciffolilli S, Murdolo G, Piroddi M, Papa S, Reiter RJ, Galli F (2014). Melatonin regulates mesenchymal stem cell differentiation: a review. J Pineal Res.

[51] Soliman A, Lacasse AA, Lanoix D, Sagrillo-Fagundes L, Boulard V, Vaillancourt C (2015). Placental melatonin system is present throughout pregnancy and regulates villous trophoblast differentiation. J Pineal Res.

[52] Kuklina EM (2014). Melatonin as potential inducer of Th17 cell differentiation. Med Hypotheses.

[53] Matsui T, Sashihara S, Oh Y, Waxman SG (1995). An orphan nuclear receptor, mROR alpha, and its spatial expression in adult mouse brain. Brain Res Mol Brain Res.

[54] Casado-Zapico S, Martin V, Garcia-Santos G, Rodriguez-Blanco J, Sanchez-Sanchez AM, Luno E, Suarez C, Garcia-Pedrero JM, Menendez ST, Antolin I, Rodriguez C (2011). Regulation of the expression of death receptors and their ligands by melatonin in haematological cancer cell lines and in leukaemia cells from patients. J Pineal Res.

[55] Osseni RA, Rat P, Bogdan A, Warnet JM, Touitou Y (2000). Evidence of prooxidant and antioxidant action of melatonin on human liver cell line HepG2. Life Sci.

[56] Tsujimoto Y, Shimizu S (2007). Role of the mitochondrial membrane permeability transition in cell death. Apoptosis.

[57] Zhang HM, Zhang Y, Zhang BX (2011). The role of mitochondrial complex III in melatonin-induced ROS production in cultured mesangial cells. J Pineal Res.

[58] Leja-Szpak A, Jaworek J, Pierzchalski P, Reiter RJ (2010). Melatonin induces pro-apoptotic signaling pathway in human pancreatic carcinoma cells (PANC-1). J Pineal Res.

[59] Martin-Renedo J, Mauriz JL, Jorquera F, Ruiz-Andres O, Gonzalez P, Gonzalez-Gallego J (2008). Melatonin induces cell cycle arrest and apoptosis in hepatocarcinoma HepG2 cell line. J Pineal Res.

[60] Trubiani O, Recchioni R, Moroni F, Pizzicannella J, Caputi S, Di Primio R (2005). Melatonin provokes cell death in human B-lymphoma cells by mitochondrial-dependent apoptotic pathway activation. J Pineal Res.

[61] Daugas E, Nochy D, Ravagnan L, Loeffler M, Susin SA, Zamzami N, Kroemer G (2000). Apoptosis-inducing factor (AIF): a ubiquitous mitochondrial oxidoreductase involved in apoptosis. FEBS Lett.

[62] Wang BQ, Yang QH, Xu RK, Xu JN (2013). Elevated levels of mitochonrial respiratory complexes activities and ATP production in 17-beta-estradiol-induced prolactin-secretory tumor cells in male rats are inhibited by melatonin *in vivo* and *in vitro*. Chin Med J (Engl).

[63] Fan J, Kang HB, Shan C, Elf S, Lin R, Xie J, Gu TL, Aguiar M, Lonning S, Chung TW, Arellano M, Khoury HJ, Shin DM, Khuri FR, Boggon TJ, Kang S (2014). Tyr-301 phosphorylation inhibits pyruvate dehydrogenase by blocking substrate binding and promotes the Warburg effect. J Biol Chem.

[64] Liu Z, Chen X, Wang Y, Peng H, Jing Y, Zhang H (2014). PDK4 protein promotes tumorigenesis through activation of cAMP-response element-binding protein (CREB)-Ras homolog enriched in brain (RHEB)-mTORC1 signaling cascade. J Biol Chem.

[65] Sharman EH, Bondy SC, Sharman KG, Lahiri D, Cotman CW, Perreau VM (2007). Effects of melatonin and age on gene expression in mouse CNS using microarray analysis. Neurochem Int.

[66] Cucina A, Proietti S, D'Anselmi F, Coluccia P, Dinicola S, Frati L, Bizzarri M (2009). Evidence for a biphasic apoptotic pathway induced by melatonin in MCF-7 breast cancer cells. J Pineal Res.

[67] Joza N, Susin SA, Daugas E, Stanford WL, Cho SK, Li CY, Sasaki T, Elia AJ, Cheng HY, Ravagnan L, Ferri KF, Zamzami N, Wakeham A, Hakem R, Yoshida H, Kong YY (2001). Essential role of the mitochondrial apoptosis-inducing factor in programmed cell death. Nature.

[68] Skehan P, Storeng R, Scudiero D, Monks A, McMahon J, Vistica D, Warren JT, Bokesch H, Kenney S, Boyd MR (1990). New colorimetric cytotoxicity assay for anticancer-drug screening. J Natl Cancer Inst.

[69] Chou TC (2010). Drug combination studies and their synergy quantification using the Chou-Talalay method. Cancer Res.

[70] Chou TC, Martin N (2007). CompuSyn Software for Drug Combinations and for General Dose-Effect Analysis, and User's Guide.

[71] Rasmussen HN, Rasmussen UF (2003). Oxygen solubilities of media used in electrochemical respiration measurements. Anal Biochem.

