# Supplementary material for: Melatonin antiproliferative effects require active mitochondrial function in embryonal carcinoma cells
Source: Oncotarget. 2015 May 20;6(19):17081–96. doi: 10.18632/oncotarget.4012 (PMC4627293; doi:10.18632/oncotarget.4012)
Supplement: Supplementary file 1 [file oncotarget-06-17081-s001.pdf]

SUPPLEMENTARY MATERIALS AND METHODS

Cell morphology and immunocytochemistry

P19 cells grown in high glucose- and galactose (glucose-free)-containing media were seeded on glass coverslips in 6-well plates for morphological studies. After completing 4-days of differentiation with retinoic acid, cell morphology was investigated by using a phase contrast light microscope (Olympus CKX4,1 Hamburg, Germany).

For immunocytochemical analysis, the incubation media were removed and cells were fixed in 4% formaldehyde in PBS during 15 min at 37ºC. After washing three times with PBS for 5 min, cells were permeabilized with 0.2% Triton X-100 in PBS during 10 min. Cells were washed again three times in PBS, incubated in PBS with 1% BSA during 1 h at 4ºC, probed with an specific primary antibody against AIF (sc-13116; Santa Cruz Biotechnology, Santa Crus, CA, USA) in PBS with 1% BSA for 2 h at 37ºC and incubated with a dilution (1:1000 in PBS with 1% BSA) of the corresponding fluorescence-conjugated secondary antibody for 1 h at 37ºC. Cells were rinsed 3 times with PBS for 5 min between labeling with primary and secondary antibodies. After labeling, coverslips were mounted on glass slides in Prolong Gold antifade medium with DAPI (P36935; Invitrogen, Paisley, UK) and cells were imaged by confocal microscopy (Zeiss LSM 510Meta, Germany). Images were obtained using LSM software and were imported and incorporated into electronic figures by using Image J and MS PowerPoint 2010.

SUPPLEMENTARY FIGURES

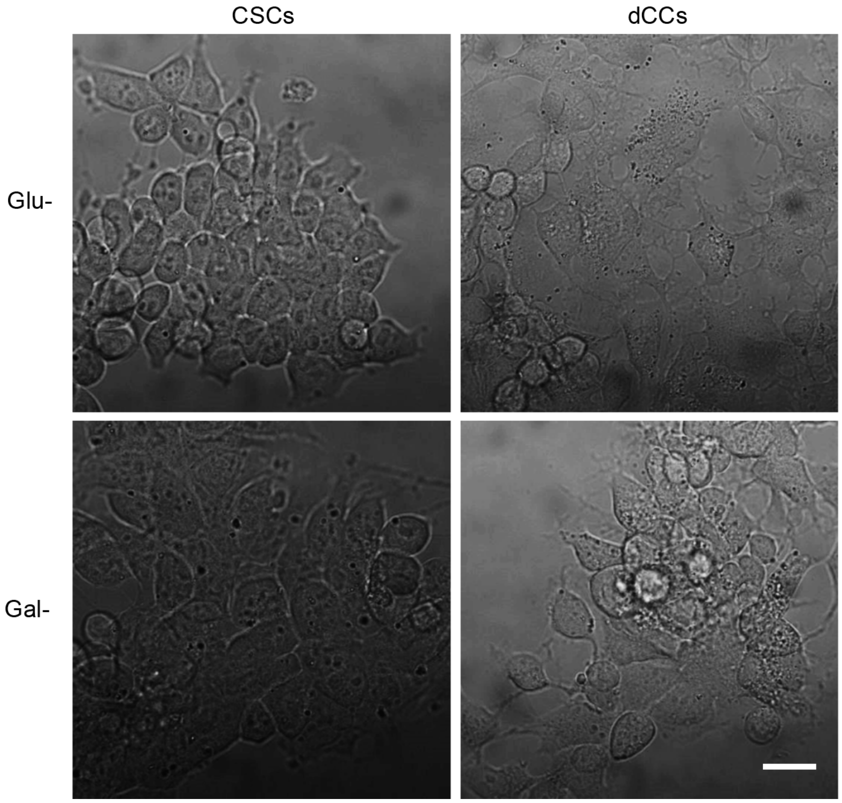

Supplementary Figure S1: Phase contrast images of P19 embryonal carcinoma stem (CSCs) and differentiated (dCCs) cells, grown in glucose (Glu) and galactose (Gal) media. Glu-CSCs exhibited visible nucleoli, round nuclei, high nucleus-to-cytoplasm ratio and a homogeneous appearance. Cell differentiation induced by the treatment with retinoic acid (dCCs) or by growing the cells in galactose-containing medium (Gal-CSCs) resulted in flattening of cells and more visible cytoplasm per nucleus which became lobular and euchromatic as compared to Glu-CSCs. All differentiated groups show a higher cellular heterogeneity, especially in Gal-dCCs which seems to present some cells with morphological alterations and incomplete differentiation. Scale bar: 20 µm.

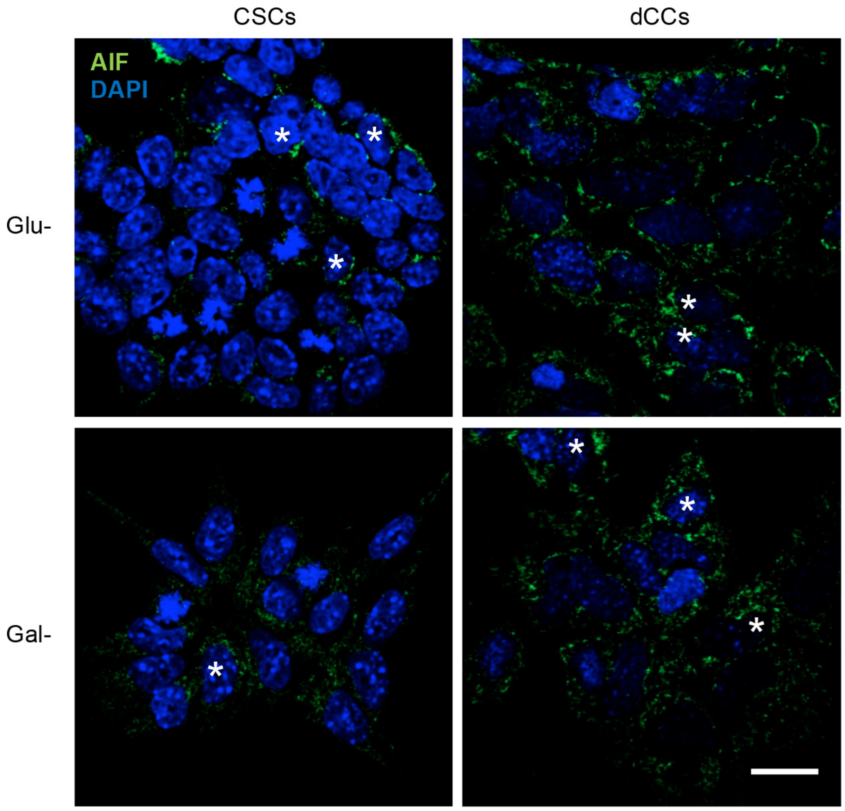

Supplementary Figure S2: Confocal images of P19 embryonal carcinoma stem (CSCs) and differentiated (dCCs) cells, grown in glucose (Glu) and galactose (Gal) media stained with DAPI (blue) and anti-AIF antibody (FITC-green) showing different cell subpopulations with dissimilar degree of AIF expression. Some cells showed higher AIF expression (asterisks). Scale bar: 20 μm.
